# Total Destruction of One Lung

**Published:** 1876-02

**Authors:** 


Total Destruction of one Lung.—Dr. J. Dowling, of Tipperary, reports the case of a girl aged eighteen, who until within a few weeks of her death had been a domestic with a farmer, who, she stated, knocked her down and kicked her. To this she attributed her illness, which was characterized by a "dragging" pain in the side of the chest, lassitude and debility. Her case was diagnosticated as one of pleuritic effusion, and after some time she was sent to hospital. She was then able to walk about the ward, but with a languid air and a feeble step. For two or three weeks before death she was confined to her bed; dyspnœa increased, her face became somewhat livid, and she lay upon her back. On post-mortem examination it was found that the right lung had been entirely destroyed, and the space formerly occupied by it was filled with purulent fluid. The former connection of the lung with the heart was marked by a semi-cartilaginous substance not more than two ounces in weight. Some serous effusion was found in the left pleural cavity, but the lung appeared healthy. The heart was of normal size. There was no fracture of the ribs, nor were there any marks of violence on the girl when she was first seen by her physician. Further investigation was prevented. The case is interesting from the fact that life was maintained until all but the whole of one lung was converted into pus, which was confined in the thorax; and also that no hemorrhage had taken place, while death from sudden hemorrhage might have been anticipated. The latter fact, Dr. D. thinks, seems to show that the process of suppuration is sufficient to occlude even vessels of the largest calibre.—*British Medical Journal.*

